# Evaluation of nutritional values, phenolic profile, aroma compounds and biological properties of *Pittosporum tobira* seeds

**DOI:** 10.1186/s12944-017-0596-1

**Published:** 2017-10-30

**Authors:** Ilhem Rjeibi, Sana Ncib, Anouar Ben Saad, Sami Souid

**Affiliations:** 1 Research unit of Macromolecular Biochemistry and Genetic, Faculty of Sciences of Gafsa, 2112 Gafsa, Tunisia; 2 Common Services Unit for Research, Faculty of Sciences of Gafsa, 2112 Gafsa, Tunisia

**Keywords:** *Pittosporum tobira* Seeds, Phenolic compounds, Aroma compounds, HS-SPME-GC-MS, Antioxidant activity, Anti-hemolytic activity

## Abstract

**Background:**

Plant essential oils and phenolic compounds are widely used for their medicinal properties. Thus, the aim of this study is to evaluate the nutritional values, the chemical composition, antioxidant activity and anti-hemolytic effects of *Pittosporum tobira* seeds.

**Methods:**

The aroma compounds were isolated using two methods (Headspace-solid phase microextraction (HS-SPME) and hydrodistillation (HD)) and analyzed by gas chromatography coupled with mass spectrometry (GC-MS). Bioactive phenolic compounds were identified by mean of high-performance liquid chromatography (HPLC-DAD). Reducing power, hydrogen peroxide (H_2_O_2_) scavenging and 2,2-diphenyl-1-picrylhydrazyl (DPPH) radical scavenging assays were used to investigate antioxidant activity. Anti-hemolytic activity was evaluated using H_2_O_2_-induced hemolysis of red blood cells (RBC).

**Results:**

Oxygenated sesquiterpenes, sesquiterpene hydrocarbons and oxygenated monoterpenes were the most volatile fractions identified by HD and HS-SPME coupled to GC-MS but their quality and amount were quite different according to the extraction methodology. The main phenolic compounds identified by HPLC were caffeic acid, followed by cinnamic acid and gallic acid. *P. tobira* seeds essential oils showed significant antioxidant activity in DPPH (IC_50_ value = 1.5 mg/mL), H_2_O_2_ scavenging assay (IC_50_ value = 159.43 μg/mL) and reducing power test (IC_50_ value = 0.982 mg/mL) compared to methanolic extract. Moreover, the results revealed that the essential oil was able to protect RBC from hemolysis induced by H_2_O_2_. However, the methanolic extract had no effect on H_2_O_2_-induced hemolysis of RBC as compared to the essential oil and the standard vitamin C.

**Conclusions:**

*P. tobira* may be used as a new natural source of antioxidant with therapeutic application in diseases caused by reactive oxygen species.

**Graphical Abstract:**

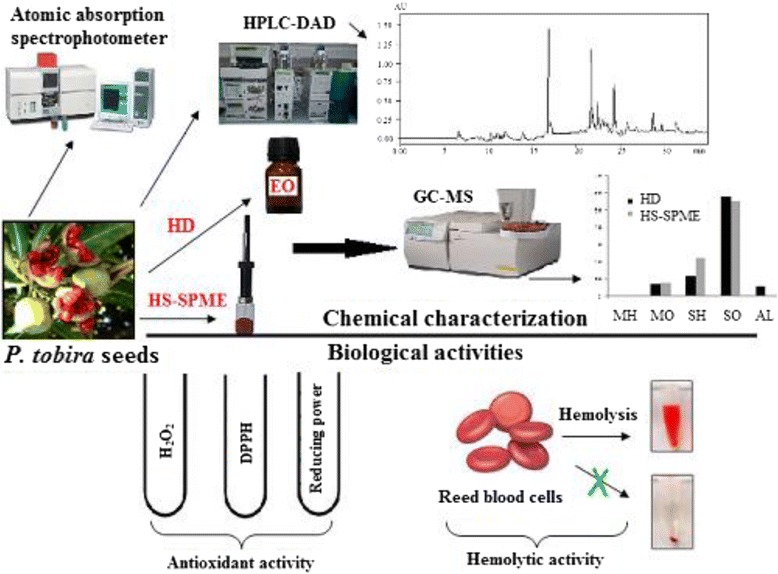
Phytochemical Characterization and Biological Evaluation of Pittosporum tobira seeds

## Introduction

All over the world, plants are known as a source of nutrients, flavoring additives, oxygen, decoration and biologically active components. The curing effects of plants derived from bioactive substances that are named secondary metabolites which include phenolic acids, flavonoids, terpenoids, tannins, coumarins and other metabolites. These compounds can be synthesized by different plant parts (leaf, root, fruit, flower and stem bark). These metabolites can exert many biological effects including anti-thrombogenic, antimicrobial, antidiabetic, hepatoprotective, antifungal and antioxidant proprieties [1].

Natural products have been found to have the ability to prevent damage caused by reactive oxygen species (ROS). These free radicals have been associated with various diseases, such as cardiovascular, liver injury, atherosclerosis, and cancer diseases [2]. In addition, ROS have been implicated in DNA mutations, lipid peroxidation and protein damage [3]. Therefore, many researchers have intensified search to characterize new antioxidant compounds from plant sources usable for clinical applications [4].

The genus *Pittosporum* forms part of the Pittosporaceae family and includes 200 species which are distributed in the temperate and hot zone of the Earth. *Pittosporum* species have been used in folk medicine of many countries in the world. *P. glabratum* from Chine has been used for the treatment of hypertension [5] and the bark of *P. neelgherrense* as antivenom [6]. *P. undulatum* from Portugal has been used to repair muscles [7]. Australian people used *P. phylliraeoides* to treat sprains and eczema [8]. This genus provides an excellent source of essential oil components such as monoterpenes, aliphatic hydrocarbons, sesquiterpenes among others compounds. Plant *P. tobira* discovered by the Europeans, is about 2–3 m high, the leaves are dark green, flowers have a smell similar to orange flowers and the black seeds are enclosed within the encapsulated fruits. Previous studies on the composition of *P. tobira* essential oils obtained by hydrodistillation procedure, have indicated the presence of *α*-pinene as the major component of the flowers for species growing in Iran [9]. This plant has been demonstrated to possess many pharmaceutical properties. Moon and Park [10], reported its protective effects against glutamate-induced neurotoxicity. Moreover, El Dib et al. [11], determined that the n-butanol fraction from *P. tobira* leaves possess antimicrobial activity and cytoprotective effects against breast carcinoma, hepatocellular carcinoma and colon carcinoma cancer cell lines. However, data on the antioxidant activities of seed essential oils from this plant are insufficient. Hence, the aim of the present research is to determine nutritional value, phenolic compound and biological activities of *P. tobira* methanolic extract. The aroma compounds composition of *P. tobira* seeds were also identified by headspace solid phase microextraction and hydrodistillation coupled to gas chromatography coupled with mass spectrometry and their antioxidant and anti-hemolytic capacities were studied.

## Materials and methods

### Plant collection

*P. tobira* seeds were sampled in June 2015 from Gafsa, southwestern Tunisia (34°25′ N and 8°47′ E). Voucher samples are stored in the herbarium of the Faculty of Sciences, University of Gafsa, Tunisia. The plant material (200 g) was allowed to air-dry at ambient temperature, grounded to a fine powder using an electric grinder and then kept at −20 °C until use.

### Physicochemical composition of *P. tobira* seeds

Moisture, protein, fat, and ash were determined using the AOAC process (1990) [12]. The ash content was determined after heat treatment at 600 ± 15 °C. Total carbohydrates have been calculated by removing from 100% the amount of moisture, total fat, protein and ash. Energy has been calculated using this equation: Energy (kcal) = 4 × (g protein + g carbohydrate) + 9 × (g fat). Mineral elements analyses were performed using the method of Rjeibi et al. [13].

### Hydrodistillation (HD)

The essential oil of *P. tobira* seeds was extracted by HD using a Clevenger-type apparatus. Briefly, 50 g of fine powder of *P. tobira* were immersed in 500 mL of distilled water and extracted during 3 h. The distilled essential oils were separated using hexane, dried over anhydrous sodium sulfate and kept in a dark glass bottle at 4 °C until further analysis.

### Headspace- solid phase microextraction (HS-SPME)

HS-SPME technique was used to identify aroma compounds from *P. tobira*. The SPME fiber 75-μm carboxen/ polydimethylsiloxane (CAR/PDMS) (preconditioned for 6 min at 240 °C) was used for analysis. The dried powdered seeds (300 mg) were hermetically sealed in 4 ml vial. The SPME fiber holder was then introduced into the vial. Both sample and fiber were incubated for 30 min at 60 °C as predefined extraction time and temperature, respectively. After extraction, the fiber was withdrawn from the sample and thermally introduced into the injection port of the GC–MS for desorption for 3 min at 240 °C in splitless mode. The experiment was performed in triplicate. The alkane solution (C_7_–C_30_ in n-hexane) performed by headspace extraction during 30 min, at 60 °C, was used to calculate the retention indices.

### GC mass spectrometry (GC-MS) analyses

The Varian CP-3800 GC equipped with the CP-8400 autosampler and Saturn 2200 mass spectrometer was used to analyze the essential oil of *P. tobira*. The injector and detector temperatures were set at 250 and 290 °C, respectively. The VF-5MS capillary column was about 30 m × 0.25 mm; 0.25 μm film thickness and the column temperature was programmed from 50 to 280 °C at 5 °C/ min and the flow rate of the carrier gas (Helium) was 1.0 mL/ min. A sample of 1.0 μL was injected, using splitless mode. Mass spectrometry was in ionization energy mode at 70 eV (scan range 40–450 m/z). Retention indices were determined by injection of the samples under the same condition of series of n-alkanes (C_7_–C_30_) and were calculated according to the equation of Dong et al. [14]. Identification of components was done by comparison of their retention times with those of pure molecules purchased from Sigma–Aldrich and by comparing their mass spectra with our own library of spectra built from pure essential oils constituents, NIST library data of the GC-MS system and from published sources.

### Methanolic extracts

50 g of the fine grounded seeds were extracted with 500 mL of methanol (80%) for 24 h at room temperature with magnetic stirring. The extracts were centrifuged at 4500×*g* for 10 min and lyophilized. All of the extracts were kept in the dark at 4 °C until use.

### Determination of total phenolic content

The total phenolic content (TPC) was determined using a modified colorimetric Folin-Ciocalteu method previously reported by Tlili et al. [15]. Briefly, Folin-Ciocalteu reagent was added to 1 mg of different sample and incubated for 5 min at room temperature. Next, 7.5% of Na_2_CO_3_ was added to the mixture and re-incubate for 60 min at room temperature in the dark. Finally, the absorbance was measured at 760 nm in a UV-Vis spectrophotometer (Shimadzu, 1240 model, Tokyo, Japan). The analysis was performed in triplicate and TPC was expressed as gallic acid equivalents in milligrams per gram of dry weight basis (mg GAE/g DW).

### Determination of total flavonoid content

The total flavonoids content (TFC) was performed according to the colorimetric assay previously published [15]. One mL of the sample (1 mg/mL) was mixed with 0.75 mL of 5% sodium nitrite solution. After 5 min, 0.15 ml of 10% aluminium chloride solution (AlCl_3_) was added and the mixture was left standing for 5 min, and then 0.5 ml of 1 M sodium hydroxide (NaOH) was added to the solution. The volume of the mixture was adjusted to 2.5 mL with distilled water and mixed well. The absorbance was measured at 510 nm. TFC was expressed as milligrams of catechin equivalent per gram dry weight basis (mg CAE/g DW).

### HPLC analysis

The analyses were performed in high-performance liquid chromatography (HPLC-DAD) with a Varian ProStar HPLC System (Varian 330/ Vis Detector and Varian 230 SDM) and C18 column (Zorbax, 4.6 mm × 250 mm). The mobile phase consisted of acetic acid (solvent A) and methanol (solvent B). The gradient was composed of 0% (B) for 2 min; 50% (B) until 30 min and 80% (B) for 5 min. The methanolic extracts were utilized in the concentration of 1 mg/mL. The flow rate was 0.9 mL/min and the volume injected was 40 μL. The detected compounds were identified by comparing their retention time with those of injected authentic standards from Sigma-Aldrich and the use of DAD spectra (200–600 nm).

### Antioxidant activity

#### DPPH radical scavenging activity

The effect of *P. tobira* seed extract and essential oil on DPPH radical was determined following the method reported by Bounatirou, [16]. Adequate solutions of each extract were created to obtain a final volume of 1 mL and were mixed with 2 mL of a freshly prepared DPPH solution (0.1 mM). The After 30 min of incubation in the dark. The absorbance was measured at 517 nm on UV–VIS spectrophotometer. Vitamin C and BHT were used as a control. Different concentrations (0.01–10 mg/mL) of the sample were used in order to determine IC_50_ (amount of sample providing 50% of scavenging on DPPH). IC50 value was determined from the linear regression equation obtained from the concentrations of the sample and the percentage of inhibition.

$$ \mathrm{I}\%=\left({\mathrm{Absorbance}}_{\mathrm{control}}-{\mathrm{Absorbance}}_{\mathrm{sample}}\right)/{\mathrm{Absorbance}}_{\mathrm{control}}\times 100. $$

#### Hydrogen peroxide (H_2_O_2_) scavenging activity

This test was done according to the method reported by Sahreen et al. [17]. Different sample concentration (1 mL) were mixed with 2.4 mL of phosphate buffer (0.1 M, pH 7.4) and 0.6 mL of H_2_O_2_ solution (40 mM). The mixture was shaken vigorously and incubated at room temperature for 10 min vitamin C was used as positive control. The absorbance was measured at 230 nm. H_2_O_2_ scavenging activity (%) = 1− Absorbance _sample_ / Absorbance _control_ × 100.

#### Reducing power assay

The reductive potential of studied samples was determined according to the method reported by Bounatirou, [16]. Different sample concentration (1 mL) were mixed with 1 mL of phosphate buffer (0.2 mol/L, pH 6.6) and 1 mL of potassium ferricyanide K_3_Fe(CN)_6_ (10 mg/mL). After incubation at 50 °C for 20 min, 1 mL of trichloroacetic acid (10 mg/mL) was added to the mixture and centrifuged at 4000 rpm for 10 min. Finally, FeCl_3_ (10 mg/mL) was added to the upper layer and left to stand for 10 min before the measurement of the absorbance at 700 nm on UV–VIS spectrophotometer (Shimadzu UV-190). The amount of sample providing 0.5 absorbances was determined from the graphical of absorbance at 700 nm. BHT was used as the control.

#### Oxidative hemolysis inhibition assay

Methods of assessment of hemolytic activity, focus on the study of the potential damage caused by hydrogen peroxide (H_2_O_2_) in erythrocyte membranes. The anti-hemolytic activity of the essential oil was determined following the method reported by Lalitha and Selvam, [18] with modifications [13]. Whole fresh human blood from healthy person (15 mL) was collected in EDTA tubes and centrifuged for 10 min at 1000 *g* at 4 °C. The plasma was removed and obtained red blood cells (RBCs) was suspended in 10 mM PBS (pH 7.4). The erythrocytes were washed 3 times with PBS and finally re-suspended in PBS to obtain a solution at 4%. One milliliter of this suspension was mixed with different concentrations (10–100 μg/mL) of the essential oils and methanolic extract and added to 7.5 mM of H_2_O_2_ prepared in PBS. After incubation with agitation for 120 min at 37 °C, the resulting mixture was centrifuged at 1000 *g* for 5 min. Finally, the absorbance of the supernatant was measured at 540 nm on UV–VIS spectrophotometer (Shimadzu UV-190). Vitamin C was used as the reference (5–30 μg/mL).

Hemolysis inhibition (%) = (Abs_2_− Abs_1_)/(Abs_2_− Abs_0_) × 100.

Abs_0_ (control) is the absorbance of RBC suspension in PBS, Abs_1_ is the absorbance of tested samples with RBC suspension in PBS/H_2_O_2_ and Abs_2_ (induced control) is the absorbance of RBC suspension in PBS and H_2_O_2_.

### Statistical analysis

Statistical analysis was performed using the SPSS version 18.0 software. All data were analyzed using a Student’s t-test. All tests were performed in triplicate and the results are given as mean values and standard deviation.

## Results and discussion

### Chemical composition of *P. tobira* seeds

The composition in total fat, moisture, protein, carbohydrate and ash contents are shown in Table 1. Carbohydrate seems the predominant component of *P. tobira* seeds, accounting for 71.25 g/100 g DW, followed by protein with 12.54 g/100 g DW. According to Pearson [19] plant food that provides more than 12% of its calorific value from protein is considered a good source of protein. The fat yield of the seed was 5.61 g/100 g DW. This value was lower than the values reported for oily seeds such as soybean [20]. Therefore, the seed could not be classified as oily seed due to the low oil content.

**Table 1:** Proximate composition, mineral content and phytochemical composition of *Pittosporum tobira* seeds

Components	Amount
Moisture (g/100 g DW)	8.02 ± 0.10
Ash (g/100 g DW)	2.58 ± 0.01
Fat (g/100 g DW)	5.61 ± 0.02
Protein (g/100 g DW)	12.54 ± 0.04
Carbohydrate (g/100 g DW)	71.25 ± 0.8
Energy (kcal/100 g DW)	385.65 ± 5.61
Potassium (K) (mg/100 g)	723.36 ± 54
Sodium (Na) (mg/100 g)	26.01 ± 0.42
Magnesium (Mg) (mg/100 g)	367.47 ± 49
Calcium (Ca) (mg/100 g)	140 ± 25
Iron (Fe) (mg/100 g)	3.05 ± 0.03
Zinc (Zn) (mg/100 g)	2.01 ± 0.01
Manganese (Mn) (mg/100 g)	1.08 ± 0.00
Gallic acid (mg/g)	1.03 ± 0.02
Caffeic acid (mg/g)	38.57 ± 0.10
Ferulic acid (mg/g)	20.07 ± 0.08
*p-*coumaric acid (mg/g)	12.85 ± 0.11
Cinnamic acid (mg/g)	5.14 ± 0.01

Values are means ± SD of three separate experiments

The seeds showed a considerable amount of ash (2.58 g/100 g DW) that may indicate the presence of considerable amounts of inorganic nutrients in this plant. All these values were in the range usually found in some cereals including quinoa, amaranth, purple corn and rice [21].

The energetic contribution of *Pittosporum* seed was also important (385.65 kcal/100 g DW). The mineral content of the *Pittosporum* seeds have been found in 2.58 g/100 g DW ash content. Also, it is important to investigate the composition of minerals in plants because they have a basic role in our diet. The composition of macroelements (K, Mg, Ca, Na) and microelements (Fe, Zn, Mn) in seeds of *P. tobira* is detailed in Table 1. As can be seen, potassium (an essential nutrient for the synthesis of proteins) was found to be the major nutrients detected in the seeds followed by Mg > Ca > Na. Ca intake helps to prevent and treat a variety of bone-related illnesses, such as osteoporosis [22]. The predominant microelement in the seed sample was Fe (3.05 g/100 g DW). The content of minerals presents similar values to those reported for other cereals and pseudocereals [21]. Moreover, food consumed by the citizens of underdeveloped countries are poor in important elements, namely Fe and Zn, and increased consumption of *P. tobira* might be useful to reward their daily needs [23].

As we can see, the consumption of *P. tobira* could cover nutritional requirements like quinoa, rice, purple corn, and amaranth. It is demonstrated that such nutraceutical and functional food ingredients have a physiological benefit and provide protection against many chronic diseases. Furthermore, they can exert a role in controlling lipid metabolism and the prevention of dyslipidaemia both in animal models and in humans [24].

### Phytochemical analysis

As shown in this study, the seeds of *P. tobira* are a rich source of phenolic compounds (Table 1). The total phenolic content was 102.7 mg GAE /g, while total flavonoid content was 31.62 mg CAE/g DW. These results corroborate previous works which described important polyphenol contents in roots, stem barks and leaves of *Pittosporum* species [8, 25]. Although the TPC values for *P. tobira* in the present study were lower compared to the ethyl acetate extract of *P. mannii* (314 mg GAE/g) .

**Fig. 1:**
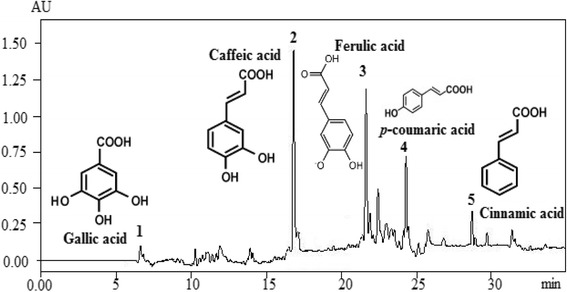
Chromatogram of the methanolic extract of *Pittosporum tobira* seeds. 1 correspond to gallic acid peak, 2 caffeic acid, 3 ferulic acid, 4 *p*-coumaric acid and 5 cinnamic acid

To our best of knowledge, there have been no studies dealing with the identification of polyphenols from *P. tobira.* The HPLC analysis of *P. tobira* seeds recorded at 280 nm revealed the presence of five phenolic acids (Fig. 1). Phenolic acids were characterized by the predominance of caffeic acid (38.57 mg/g) followed by ferulic acid (20.07 mg/g), *p-*coumaric acid (12.85 mg/g), cinnamic acid (5.14 mg/g) and gallic acid (1.03 mg/g) (Table 1). Phenolic compounds that have been detected in *P. angustifolium* include quercetin glycosides rutin, isoquercitrin dicaffeoylquinic acid [26]. It has been reported that such compounds exhibit diverse therapeutic and biological properties that may justify the use of *P. tobira* in traditional medicine.

### Analysis of the aroma compounds of *P. tobira* by HD and HS-SPME

To our knowledge this study is the first report on the extraction of aroma compounds from *P. tobira* using both HD and HS-SPME methods. The yield of essential oils obtained by HD was 0.84%. A total of 19 components representing 89.494% of the total essential oils were identified (Table 2). The essential oil of *P. tobira* seeds was characterized mainly by oxygenated sesquiterpenes (57.517%) followed by sesquiterpene hydrocarbons (11.794%) and oxygenated monoterpenes (6.659%). The most representative components were spathulenol (47.989%), isospathulenol (5.798%), *δ*-Elemene (4.195%), *λ*-gurjunene (4.034%) and camphor (3.657%). The presence of sesquiterpenes as a major volatile component in the leave from other species such as *P. viridulum,* have been already reported by John et al. [6]. According to [27] Myrcene and n-Nonane were the main components of the essential oil from the flowers and leaves of *P. tobira*, respectively.

**Table 2:** Chemical composition (GC–MS) of *Pittosporum tobira* seed obtained by HD and HS-SPME

Compounds ^*^	RI^a^	% RC		Molecular formula	Identification methods
		SPME	HD		
1-Butanol	862	–	3.057	C_4_H_10_O	MS,RI
3-Carene	1007	–	0.357	C_10_H_16_	MS,RI
Eucalyptol	1020	–	1.901	C_10_H_18_O	MS, RI
Artemisia alcohol	1083	–	0.119	C_10_H_18_O	MS, RI
Camphor	1125	7.824	3.657	C_10_H_16_O	MS, RI, Co-GC
Borneol	1165	–	2.62	C_10_H_18_O	MS,RI
D-Verbenone	1185	–	1.101	C_10_H_14_O	MS, RI
δ-Elemene	1332	0.412	4.195	C_15_H_24_	MS, RI
Eugenol	1340	0.956	2.792	C_10_H_12_O_2_	MS, RI
*&*-Cubebene	1351	0.339	–	C_15_H_24_	MS, RI
*&*-Copaene	1375	1.724	–	C_15_H_24_	MS, RI, Co-GC
*β*-Elemene	1388	1.257	–	C_15_H_24_	MS, RI
*β*-Caryophyllene	1418	1.047	0.773	C_15_H_24_	MS, RI
*β*- Copaene	1427	0.873	–	C_15_H_24_	MS, RI
*&*-Patchoulene	1452	0.337	–	C_15_H_24_	MS, RI
λ-Gurjunene	1467	2.068	4.034	C_15_H_24_	MS, RI, Co-GC
Himachalene	1471	10.493	–	C_15_H_24_	MS, RI
Germacrene D	1477	0.967	–	C_15_H_24_	MS, RI
Spathulenol	1568	51.45	47.989	C_15_H_24_O	MS, RI, Co-GC
Longifolene	1576	0.372	–	C_15_H_24_	MS, RI
Isoaromadendrene epoxide	1579	0.107	1.215	C_15_H_24_O	MS, RI
Globulol	1582	0.165	–	C_15_H_26_O	MS, RI
Ledol	1585	–	0.52	C_15_H_26_O	MS, RI
Guaiol	1588	–	0.383	C_15_H_26_O	MS, RI
Cubenol	1618	0.567	–	C_15_H_26_O	MS, RI
Isospathulenol	1625	2.701	5.798	C_15_H_24_O	MS, RI
*T-*Cadinol	1633	0.135	1.612	C_15_H_26_O	MS, RI
Unknown	1821	10.747	2.036	C_15_H_24_O	MS,RI
1-Eicosanol	2219	–	5.335	C_20_H_42_O	MS,RI

^a^ *RI* retention index relative to C_7_–C_30_ n-alknes determined using on a VF-5MS capillary column. *RC* relative concentration, *MS* mass spectrum, *Co-GC* co-injection with authentic compound

**Fig. 2:**
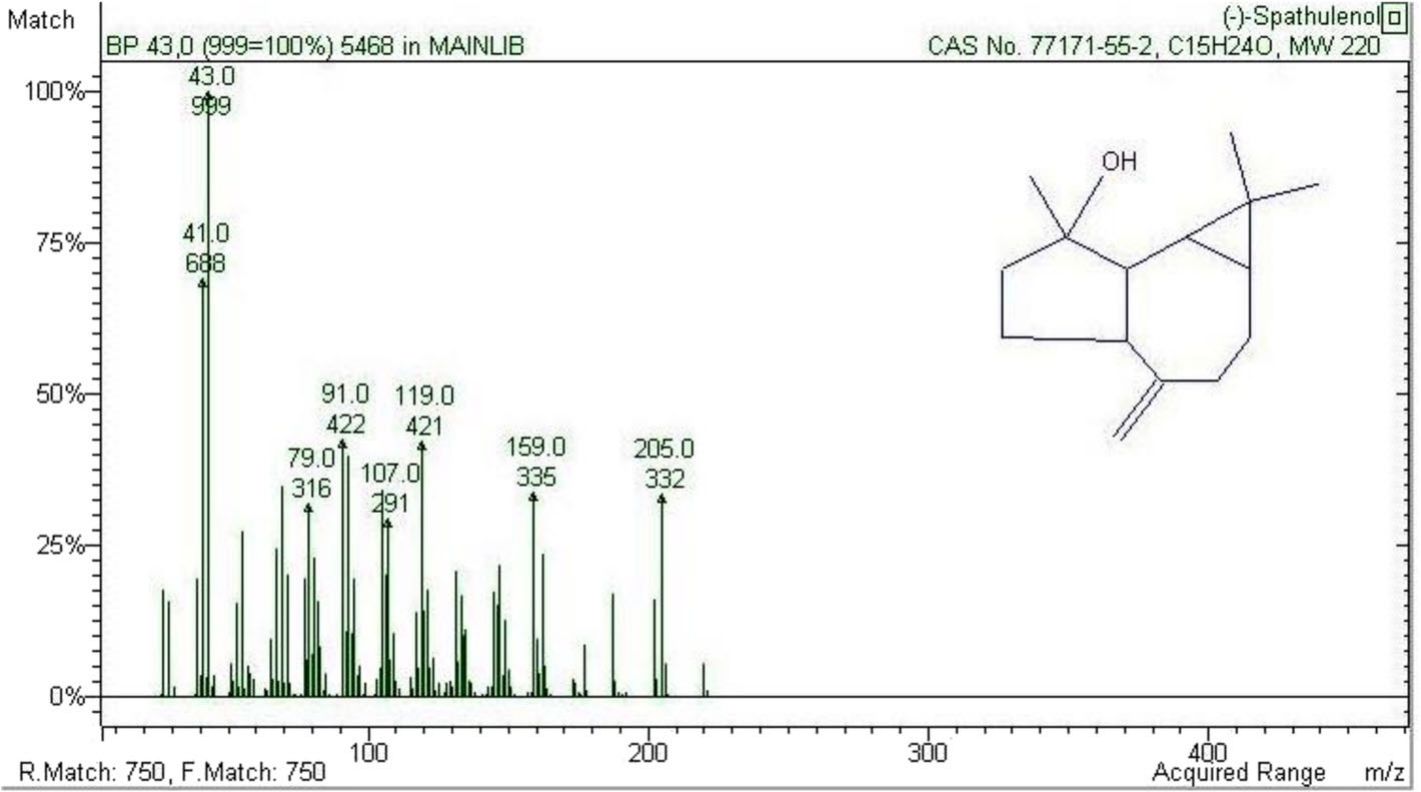
Mass spectrum and compound structure of spathulenol

GC–MS analysis of the HS-SPME aroma compounds obtained from *P. tobira* seed led to the identification of 20 components corresponding to 95.5% of the total oil content (Table 2). As already seen in HD, sesquiterpenes showed a similar pattern using SPME extraction: oxygenated sesquiterpenes were the major fraction of the essential oil (55.12%) followed by sesquiterpene hydrocarbons (21.80%) and oxygenated monoterpenes (7.82%). However, some differences in the amounts of major components were detected according to the extraction methods. As appears from Table 2, spathulenol (51.45%) was the main sesquiterpenes detectable by SPME. The mass spectrum of spathulenol and its compound structure is illustrated in Fig. 2.

As such no quantifiable data is reported on the characterization of the essential oil from *Pittosporum* species using HS-SPME with which to compare the results of our present study.

**Fig. 3:**
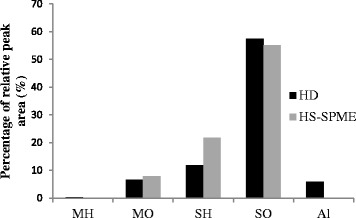
Schematic diagram representing the variations of the aroma compounds from *Pittosporum tobira* seeds identified by hydrodistillation (HD) and HS-SPME methods. Monoterpene hydrocarbons (MH), oxygenated monoterpenes (MO), sesquiterpene hydrocarbons (SH), oxygenated sesquiterpenes (SO) and alcohols (Al)

The variation of the identified volatile substances, using both methods, are given in Fig. 3. Our results clearly showed that the quality and the amount of the essential oil vary according to the extraction methodology [28]. In fact, HD and HS-SPME have allowed the detection of the majority of volatile compound in the essential oil of *P. tobira* but their proportions are dependent on the separation technique. In addition, HS-SPME allowed a better extraction of sesquiterpene hydrocarbons (21.80%) compared to the HD methods. According to Table 2, a remarkable amount of himachalene (10.493%) was detected by HS-SPME, while it was completely absent in comparison to the aromas obtained from HD. Moreover, monoterpenes hydrocarbons (0.357%) and alcohols (5.796%) were not detected using SPME extraction. It is noteworthy that the HS-SPME analysis, had favored a qualitative estimation of the volatile components using a small amount of plant. Finally, further works on the HS-SPME are needed to improve the extraction efficiency such as selection of fiber, optimization of extraction time and temperature [29].

### Antioxidant activities

**Fig. 4:**
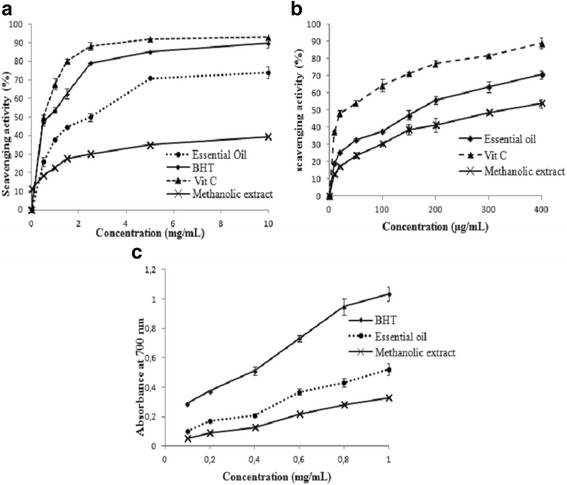
Antioxidant activities of essential oil from *Pittosporum tobira* seeds at different concentrations. DPPH free radical-scavenging activities (**a**), hydrogen peroxide (H_2_O_2_) scavenging activity (**b**) and reducing power (**c**). Values are means of three replications ± SD

As illustrated in Fig. 4a, the DPPH scavenging activity of all samples increased in a manner dependent on the concentration. At the concentration of 10 mg/mL, *P. tobira* seeds essential oil showed the highest radical scavenging activity value (89.24%) but significantly lower than the vitamin C and BHT (95.97% and 93.66%, respectively) (*p* < 0.05). More precisely, the IC_50_ value of the tested oil (1.5 ± 0.21 mg/mL) was less efficient than that of the standards BHT (0.093 ± 0.004 mg/mL) and vitamin C (0.0105 mg/mL) (Table 3).

**Table 3:** Antioxidant capacity of essential oil and methanolic extract of *Pittosporum tobira* seeds

	DPPH scavenging activity (IC_50_, mg/mL)^*^	Reducing power (EC_50_, mg/mL)^**^	(H_2_O_2_) scavenging activity (IC_50_, μg/mL)^***^	Anti-hemolytic activity (IC_50_, μg/mL)
Essential oil	1.5 ± 0.21^c,d^	0.982 ± 0.24^b^	159.43 ± 2.7^c^	116.54 ± 1.8^c^
Methanolic extract	> 10	> 1	308.57 ± 8.14^f^	–
Synthetic antioxidant				
BHT	0.093 ± 0.004^a^	0.384 ± 0.102^a^	–	–
Vit C	0.0105 ± 0.001^b^	–	20.73 ± 0.78^a^	16.75 ± 0.61^a^

Values are mean ± SD, *n* = 3 (three independent experiments)

Different letters for the same column indicate significant differences at *p* < 0.05

^*^ IC50 value was the effective concentrations at which DPPH were scavenged by 50%

^**^EC50 value: the effective concentration at which the antioxidant activity was 50%; the absorbance was 0.5 for reducing power

^***^ IC50 value was the effective concentrations at which H_2_O_2_ were scavenged by 50%

*Vit C* Vitamin C, *BHT* Butylated hydroxytoluene

As appears from Fig. 4b, antioxidant activity determined using the (H_2_O_2_) scavenging assay showed results comparable to those obtained with DPPH scavenging activity. Once again, the result suggests that *P. tobira* seeds essential oil exhibited the strongest antioxidant activity. This inhibition was dose-dependent (H_2_O_2_) scavenging ability with IC_50_ values of 159.43 ± 2.7 and 308.57 μg/mL for essential oil and methanolic extract, respectively. Thus, IC_50_ values of essential oil were 1.93-fold more efficient than the methanolic extract but less efficient than vitamin C (IC_50_ = 20.73 ± 0.78 μg/mL) (Table 3).

The reducing power assay was commonly reported as a simple and robust method for measuring antioxidant power. This test is based on the conversion of Fe^3+^ to Fe^2+^ under the effect of essential oils. In agreement with DPPH and (H_2_O_2_) scavenging assays, reducing power method showed that essential oils exhibited good antioxidant activity. Thereby, the reducing powers of the tested samples increased when their concentrations were increased (Fig. 4c). As shown in Table 3, the tested oil had slightly lower antioxidant potential than BHT (EC_50_ = 0.384 ± 0.102 mg/mL for BHT and EC_50_ = 0.982 ± 0.24 mg/mL for tested oil), but better than methanolic extract.

Overall, the results obtained from the three methods have shown that *P. tobira* exhibited an important antioxidant activity. The obtained results are in agreement with several reports. Notably, the recent study of Dong et al. [14], demonstrated that essential oil from *Chuanminshen violaceum* had potent antioxidant activity in vitro when compared to other sub-fraction like ethanol, ethyl acetate, methanol and chloroform fraction. However, findings of Mohamed et al. [30] suggested that methanolic extract of *Commiphora myrrha* exhibited higher DPPH scavenging capacity than the essential oil. These results suggested that there is a relationship between the chemical composition of aromatic plants and their antioxidant activity. According to our results, the antioxidant activity of *P. tobira* seed essential oil might be attributed to the presence of biologically active component such as spathulenol, λ-gurjunene, camphor and isospathulenol. Spathulenol was found to be the important component in many medicinal plants such as *Stachys inflata* and *Kundmannia syriaca* [31, 32]. Additionally, previous studies showed the immunomodulatory effects of spathulenol identified from *Salvia spinosa* essential oil [33]. Moreover, studies of Martins et al. [34], have demonstrated the anti-tumor potential of spathulenol.

Despite all these investigations, it is difficult to attribute the antioxidant activity to the major active compounds of the essential oil. Indeed, it is also important to take into account minor compounds for their antioxidant activities [35]. In this regard, the individual compound should be studied.

### Inhibition of human erythrocyte hemolysis

**Fig. 5:**
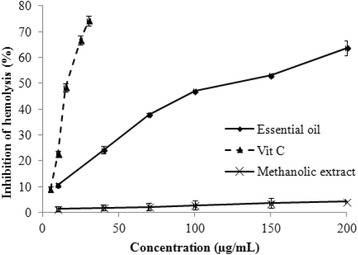
Effects of the essential oil and methanolic extract from *Pittosporum tobira* seeds on red blood cell hemolysis induced by H_2_O_2_ (7.5 mM). Values are means of three replications ± SD

The erythrocytes are the most abundant cells in human body and possess many biological characteristics enabling them to ensure drug transport. The excess of ROS can generate oxidative stress conditions that could damage the erythrocyte membrane lipids and proteins and possibly lead to hemolysis [36]. Given so, in this experimental work, we aimed for the first time to assess whether *P. tobira* prevents damages to erythrocyte membrane or not. Therefore, RBC (red blood cells) were chosen as easy biological design. As shown in Fig. 5, *P. tobira* seeds oil inhibited the hemolysis of erythrocytes in a dose-dependent pattern. The maximum of anti-hemolytic activity was observed at 63.64% with 200 μg/mL of essential oil and the IC_50_ value was 116.54 μg/mL (Table 3). Results demonstrated that essential oil protected erythrocytes from H_2_O_2_-induced hemolysis. However, the methanolic extract at the concentration from 10 μg/mL to 100 μg/mL, have no effects on RBC. Accordingly, essential oil from *P. tobira* seeds may contain more anti-hemolytic compounds as compared to methanolic extract. Moreover, the antioxidant compounds present in *P. tobira* might be responsible for the anti-hemolytic activities. These findings are in agreement with the results obtained by Miyazaki et al. [37], who reported the protective effect of natural antioxidant against the degradation of RBC membrane by ROS.

## Conclusion

This study shows, for the first time, the aroma compounds composition of *P. tobira* using hydrodistillation and HS-SPME coupled to GC-MS. Results revealed that the predominant components are sesquiterpenes and that the quality and the quantity of the essential oil vary according to the extraction methodology. This essential oil showed high antioxidant activity and significant inhibitory activity against H_2_O_2_-induced hemolysis. Our findings suggested that *P. tobira* could be considered as a novel source of natural antioxidant that may be used to protect the human body against disease caused by free radicals.

## Abbreviations

BHT: butylated hydroxytoluene
DPPH: 2, 2-diphenyl-1-picrylhydrazyl
GC/MS: gas chromatography/mass spectrometry
H_2_O_2_: hydrogen peroxide
HD: hydrodistillation
HPLC: high-performance liquid chromatography
HS-SPME: Headspace- solid phase microextraction
PBS: phosphate buffer solution
RBC: red blood cells
ROS: reactive oxygen species
